# Competitive interactions affect working memory performance for both simultaneous and sequential stimulus presentation

**DOI:** 10.1038/s41598-017-05011-x

**Published:** 2017-07-06

**Authors:** Jumana Ahmad, Garrett Swan, Howard Bowman, Brad Wyble, Anna C. Nobre, Kimron L. Shapiro, Fiona McNab

**Affiliations:** 1 School of Psychology, University of Birmingham, Birmingham, UK; 2 Department of Forensic and Neurodevelopmental Science, Kings College London, London, UK; 3 Department of Psychology, The Pennsylvania State University, Pennsylvania, USA; 4 Centre for Cognitive Neuroscience and Cognitive Systems (CCNCS), School of Computing, University of Kent, Canterbury, UK; 5 Department of Experimental Psychology and Oxford Centre for Human Brain Activity, University of Oxford, Oxford, UK; 6 Department of Psychology, University of York, York, UK

## Abstract

Competition between simultaneously presented visual stimuli lengthens reaction time and reduces both the BOLD response and neural firing. In contrast, conditions of sequential presentation have been assumed to be free from competition. Here we manipulated the spatial proximity of stimuli (Near versus Far conditions) to examine the effects of simultaneous and sequential competition on different measures of working memory (WM) for colour. With simultaneous presentation, the measure of WM precision was significantly lower for Near items, and participants reported the colour of the wrong item more often. These effects were preserved when the second stimulus immediately followed the first, disappeared when they were separated by 500 ms, and were partly recovered (evident for our measure of mis-binding but not WM precision) when the task was altered to encourage participants to maintain the sequentially presented items together in WM. Our results show, for the first time, that competition affects the measure of WM precision, and challenge the assumption that sequential presentation removes competition.

## Introduction

Objects in a visual scene compete for neural coding during perception. Single cell recordings^1–3^ and fMRI results indicate mutual suppression of items within the same receptive field (RF)^4, 5^. The effects of competitive interactions between stimuli are apparent behaviourally, with greater feature similarity between targets and distractors slowing visual search performance e.g. ref. 6.

It has been claimed that the effects of such competition extend beyond ‘perception’ to the higher-level processes of working memory (WM). Shapiro and Miller^7^ review evidence to support this assertion, citing Ihssen, Linden, and Shapiro^8^ (see also ref. 9), who reveal greater WM capacity for eight items presented in two sequential displays, compared to simultaneous presentation of all items. This suggests more items can be held in WM when competition between stimuli is reduced. Here we examine whether the effects of competition extend to WM precision. We do not address whether items compete within WM, we examine whether the effects of competitive-interactions (irrespective of the stage at which they occur) can be observed in the measure of WM precision.

Furthermore, we address whether competition operates only when both items are perceived together or whether items held in WM can also contribute to, or be subject to, the effects of competition. It is often assumed that conditions in which items are presented sequentially are free from the effects of competition e.g. refs 4 and 5. However, given the recruitment of early visual cortex for WM maintenance^10, 11^, and evidence for early visual cortex providing a cortical basis for the active maintenance of information about features and locations of stimuli^12^, it is also possible that items held in WM may compete with each other, or with new stimuli that are being encoded.

In order to address these two questions we used the ‘colour wheel’ variant of the change-detection paradigm, which enables WM precision to be estimated e.g. refs 13–15. We manipulated competition by varying the spatial proximity within which the stimuli were presented. Manipulating proximity has been shown to influence performance in tasks other than change detection, for example in multiple object identification and tracking^16–18^. Consistently, the extent of neural suppression due to competition is inversely related to the degree of spatial separation between stimuli^5^. With a sufficient distance between stimuli, the effects of competition can be eliminated^19^. Furthermore, crowding effects reveal another example of substantial interference in identifying one stimulus when other stimuli with similar features are nearby^20^.

We manipulated the spatial separation of coloured stimuli at encoding first with simultaneous presentation (Experiment 1), then with sequential presentation (Experiments 2–5). In line with others e.g. ref. 21, we interpret any difference in WM performance between conditions of high spatial separation and conditions of low spatial separation as evidence of competition.

Recent studies have begun to address whether the effects of competition extend to various measures of WM performance. Also using the “colour wheel” approach, Emrich and Ferber^21^ observed more binding errors (reporting the colour of an uncued item) for the high competition condition, but no difference in WM precision. The spatial distance between stimuli in their high competition condition (3.1°) may explain the absence of a precision difference. These stimuli were unlikely to compete strongly within primary visual cortex, where RFs are estimated to be less than 2° in monkeys^22^ and humans^5^. With our first experiment, we compared conditions of high and low spatial proximity, presenting the items in the Near condition within 0.6°, to create greater competition throughout the visual hierarchy. We examined whether the effects of competition extend to a measure of WM precision as well as binding.

Using a similar approach, in which participants reproduced the orientation of a bar presented in conditions of high or low spatial crowding, Tamber-Rosenau, Fintzi and Marois^23^ reported more binding errors (reporting the orientation of an uncued item) in the high crowding condition. This was observed for both a perceptual task in which the stimuli remained on the screen during the response phase, and a WM task in which the stimuli disappeared 800 ms prior to the response phase. In their results, the precision with which the orientation was reproduced was lower for the high crowding condition compared to the low crowding condition only for the perceptual task, not in the WM task. Again, it is possible that the stimuli presented in the high crowding condition were not close enough for the effects of competition to be observed on WM precision.

Apparently contradictory findings were provided by Lin and Luck^24^, who reported that greater colour similarity between items enhanced WM performance. Participants were required to detect changes in colour, which was also the feature used for the competition manipulation. The conflation of the competing feature (colour) and the task demand (colour report) may have accounted for Lin and Luck’s result^24^. In the current design, the feature dimension used to create competition (spatial proximity) was orthogonal to the response dimension (colour report) and the stimuli were always a fixed distance apart in colour space.

In this way, our five experiments addressed whether competition between simultaneously-presented proximal stimuli affects various measures of WM performance and whether items held within WM can also contribute to or be subject to the effects of competition.

## General Method

All five experiments were approved by the Ethical Review Committee of the University of Birmingham and were conducted in accordance with the Declaration of Helsinki. Written, informed consent was obtained from all participants.

## Experiment 1

### Method

In each trial, participants were asked to retain two simultaneously presented items in WM. In the Near condition, stimuli were positioned 0.25° apart (centre to centre) so that, according to estimates of RF size, they should compete throughout the visual hierarchy. In the Far condition, items were positioned 6.70° apart (centre to centre) such that there should be less competition in most visual areas. The subsequent WM representation for one of the items was examined by asking participants to use the mouse to indicate the colour of the probed item on a colour wheel cf. refs 13–15. WM performance was assessed using a mixture model^15^. We used two approaches. First, we adopted the established approach of estimating a separate mixture model for each spatial separation condition and comparing the resulting model parameters using ANOVA e.g. ref. 21, 23. Due to concerns that summary statistics are unreliable for model comparison^25^, we also used a non-parametric hierarchical model comparison approach in which permutation testing was used to determine whether different components of WM performance differed with spatial competition.

### Participants

Twenty-one students from the University of Birmingham participated in Experiment 1. Data from one participant were removed as they failed to follow instructions on a substantial number of trials, did not perform the phonological suppression task correctly and clicked outside the colour wheel on a large number of trials. Two participants withdrew during the experimental procedure. Data from a further participant were removed as they had a score of more than 3 standard deviations from the group mean for at least one of the WM measures. This left 17 participants (15 females, 2 males; mean age = 22 years, range = 18–32 years).

All participants were naive to the purpose of the experiment, and were compensated for their participation with course credit or £6 per hour. All had normal or corrected-to-normal vision, and passed the Farnsworth Munsell Dichotomous D15 Colour Vision Test.

### Stimuli and Design

Stimuli were displayed on a 39 cm × 29 cm CRT monitor. The screen resolution was 1024 × 768, and the refresh rate was 60 Hz. The monitor was calibrated with a *SpectroCAL Spectroradiometer* so that all stimuli had a luminance of approximately 20 cd m^−2^. A chin rest was used to maintain a viewing distance of 70 cm throughout the experiment. The stimuli were presented using MATLAB with Psychophysics Toolbox 3 extensions^26, 27^.

**Figure 1:**
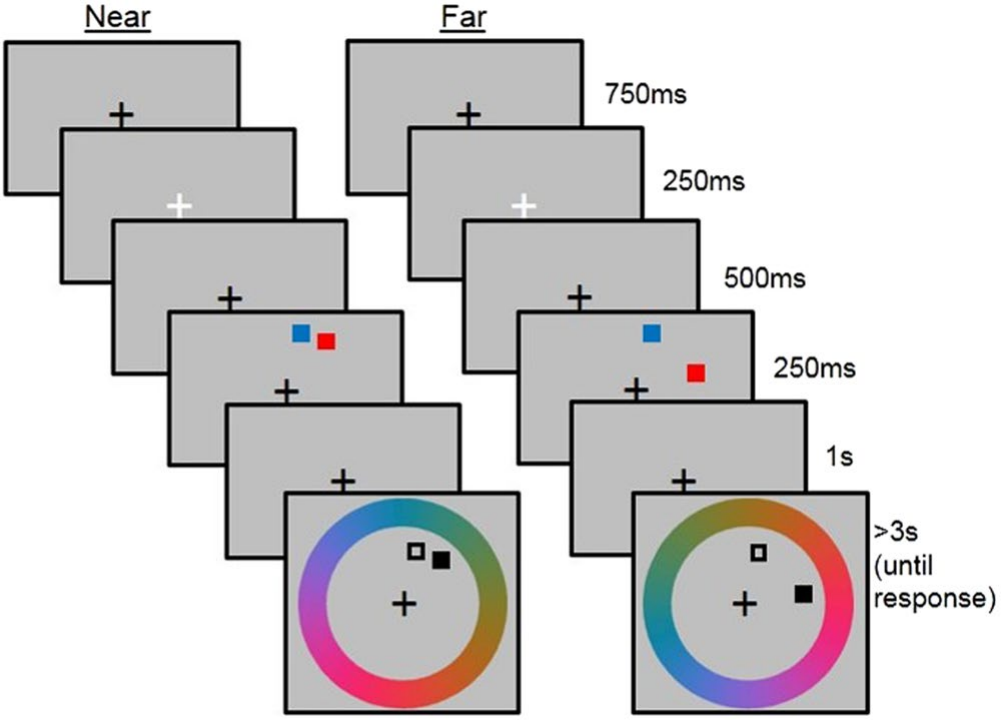
The trial sequence for the Near and Far conditions for Experiment 1.

In each trial, participants were asked to encode the colours of two squares (0.21° × 0.21°) appearing along an invisible circle at 5.50° eccentricity. Both squares always appeared in one quadrant of the screen (Fig. 1). The two squares were either 0.25° apart (centre-to-centre; contained within 0.60°; *Near* condition), or 6.70° apart (centre-to-centre; contained within 7.00°; *Far* condition). The quadrant in which the squares appeared was varied pseudo-randomly, and counterbalanced across conditions.

Participants were asked to indicate the colour of one of the squares (the target), the position of which was indicated by a black filled square. In the place of the other coloured square (the non-target) a black outline square was shown. Participants were asked to indicate the colour of the target by clicking on a colour wheel; a ring that was 1.60° thick and centred on the monitor with a radius of 9.33° (centre to outer edge). The colour wheel consisted of 180 colours, with two degrees on the wheel allocated to each colour. Due to an error in Experiment 1, the colour wheel consisted of 179 colours, with four degrees for one of the colours. Trials featuring a target stimulus of that colour, or the missing colour, were removed from the analysis (no more than three trials per condition, per participant).

The colour values were evenly distributed along a circle in the CIE L*a*b colour space, centred at (*L* = 70, *a* = 22, *b* = 13), with a radius of 60. The centre was chosen to maximise the radius and the discriminability of the colours cf. ref. 14. All colours were of equal luminance, varying in hue. The colour wheel was rotated randomly for each trial, with 10 possible positions, each separated by 36°, so that the position of the colours could not be predicted.

Each coloured square was filled with one of the colours represented in the colour wheel. Colour allocation was pseudo-random, such that for each pair of squares, the colours differed by 60 colour units (120° on the colour wheel), so that the colour distance between items was equated across all trials. Although this may have made it possible for a participant to infer the colour of a stimulus from the colour of the other stimulus, they would not have been able to infer whether the second stimulus was to 120° clockwise or 120° counter-clockwise to the first. The same colour pairs were used for stimuli in the Near and Far conditions, to avoid any confound associated with the choice of colours.

Following 30 practice trials, participants completed 640 experimental trials (half were Near trials and half were Far trials, presented in a randomised and unpredictable order). The experiment was divided into four equal length experimental blocks, separated by breaks.

### Procedure

The trial procedure and durations of presentation are shown in Fig. 1. Each trial began with a black fixation cross (presented for 750 ms), which turned white for 250 ms to prepare participants for the onset of the memory stimuli (the stimuli to be encoded). The memory stimuli (presented for 500 ms) were followed by a 1000 ms delay period, during which time a black fixation cross was displayed. A colour wheel was then shown, together with two squares in the positions that had been occupied by the memory stimuli. One of the stimuli was outlined in black and the other was filled black. Participants were asked to report the colour of the memory stimulus that had been in the position of the filled black square, by clicking on the colour wheel. The colour wheel remained on the screen until for at least 3 s, beyond which it was removed when a response was made. Responses were made using a computer mouse and either the right or left hand. Participants were told to respond as accurately as possible, and to maintain fixation in the centre of the screen until a response was required. They were not told that the stimuli would vary in their spatial separation, and no feedback of task performance was given. In order to minimise the opportunity for verbal rehearsal, participants performed a phonological suppression task throughout the experiment, whereby they repeated three random numbers out loud at an approximate rate of three every two seconds, which was monitored by the experimenter. The numbers changed at each of the three breaks.

### Data Analysis

Trials in which a participant clicked outside of the colour wheel were removed from the analysis. For each participant and each condition, the distribution of responses was analysed using a probabilistic three-component mixture model^15^ (http://www.paulbays.com/code). The contribution of each of three components was estimated using a maximum likelihood approach: target responses (*P*_T_, the probability of responding correctly), non-target responses (*P*_NT_, the probability of responding with the colour of the unprobed memory stimulus, which may result from mis-binding the colour and the location), and uniform responses (*P*_U_, the probability of a response that did not match the colour of either of the two memory stimuli, which is interpreted as a guess). From this model, the precision (K) with which items are represented can be estimated using the standard deviation of the circular analogue of Gaussian distributions around both the target and the non-target values. We did not correct for multiple comparisons because we had a priori identified the precision measure as being key to our hypothesis.

**Figure 2:**
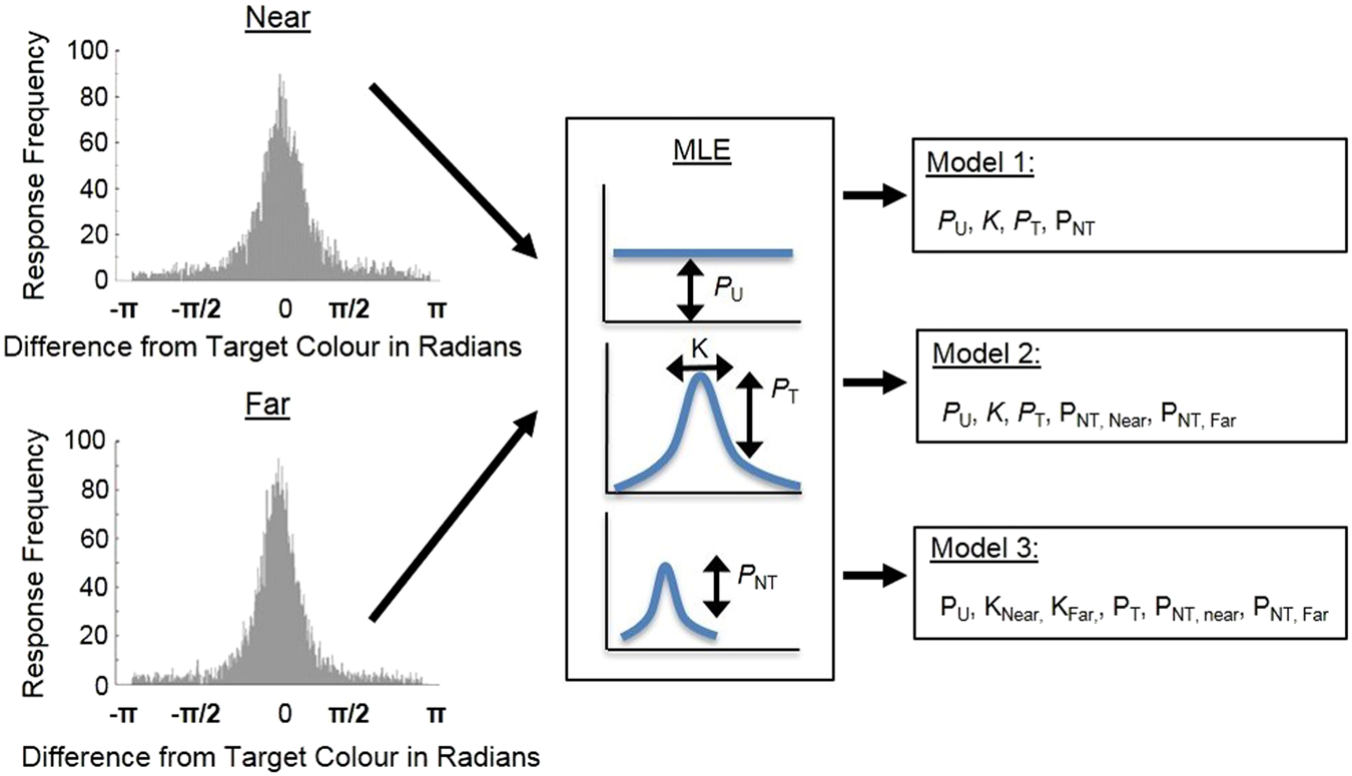
An illustration of the model comparison process. The Near and Far condition data was fit using Maximum Likelihood Estimation (MLE). The number of free parameters in the MLE is determined by the Model on the right. *P*_T_ represents the probability of responding correctly to the target, *P*_NT_ represents the probability of responding with the colour of the unprobed memory stimulus (the non-target), *P*_U_ represents the probability of a response that did not match the colour of either of the two memory stimuli, which is interpreted as a guess, K represents the precision with which items are represented. In our “null hypothesis mixture model” (Model 1), we fit the Bays *et al*. mixture model^15^ to the data from each participant and made no distinction between Near and Far conditions. For Model 2, only *P*_NT_ could vary between Near and Far conditions. For Model 3, both K and *P*_NT_ could vary between Near and Far conditions.

Response distributions for each participant were also subjected to a non-parametric hierarchical model comparison (illustrated in Fig. 2). This approach enabled us to determine whether, for example, a model in which there were separate values for precision (K) and *P*_NT_ for the Near and Far conditions provided a better fit than a model in which these parameters did not differ between the Near and Far conditions. Using hierarchical model comparison, we determined whether and how responses differed between Near and Far conditions. For each model, we used the constraint that *P*_T_ (the probability of correctly reporting the target), *P*_NT_ (the probability of reporting the non-target), and *P*_U_ (the probability of guessing) sum to one. In our “null hypothesis mixture model” (Model 1), we fit the mixture model^15^ to the data from each participant and made no distinction between Near and Far conditions, i.e., one set of parameter values was used irrespective of condition and participant. We then compared this model to alternative models, which allowed the proportion of non-target responses and precision to vary for the Near and Far conditions.

## Results

**Figure 3:**
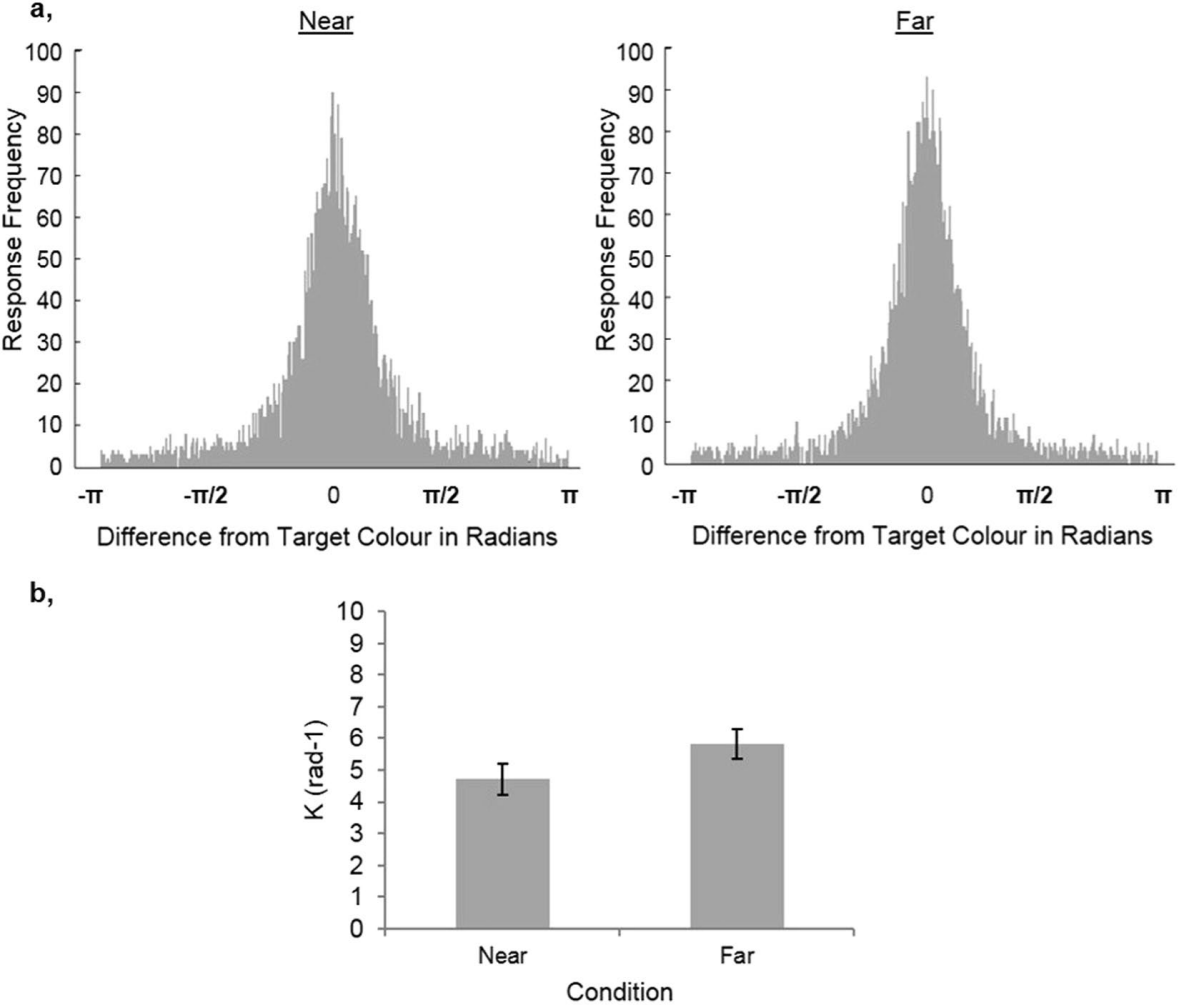
The results of Experiment 1. (**a**) The distribution of responses relative to the target colour (shown at 0 radians) for the Near and Far conditions, pooled across participants. (**b**) The mean precision estimates (K) for each condition, calculated using the mixture model outlined by Bays, Catalao, & Husain^15^. Error bars indicate the standard error of the mean. *Indicates p < 0.05 for the paired t-test between Near and Far conditions.

For each condition, the distribution of responses, relative to the target colour, is shown in Fig. 3a.

### Precision

Mean estimates of precision are shown for each condition in Fig. 3b. In line with our prediction, precision was lower for the Near compared to the Far condition (paired t-test *t*(16) = −2.85, *p* = 0.012, Cohen’s d: −0.693).

### *P*_T_, *P*_NT_ and *P*_U_

Estimates for the probability parameters, *P*_T_, *P*_NT_, and *P*_U_ are shown in Table 1.

**Table 1:** Estimates from the mixture model for all experiments, with the standard deviation shown in parentheses. The results of paired t-tests between Near and Far conditions are shown. *Represents *p* < 0.05, **represents *p* < 0.01.

	Condition	K	*P*_T_	*P*_NT_	*P*_U_
Experiment 1	Near	4.71 (2.02)	0.83 (0.09)	0.08 (0.07)**	0.08 (0.07)*
	Far	5.81 (1.92)	0.84 (0.09)	0.03 (0.03)	0.13 (0.08)
Experiment 2	Near 1^st^	5.00 (1.62)	0.79 (0.11)**	0.13 (0.13)**	0.08 (0.08)
	Far 1^st^	6.39 (2.57)	0.84 (0.10)	0.06 (0.09)	0.09 (0.07)
	Near 2^nd^	5.67 (2.87)	0.84 (0.10)	0.08 (0.07)	0.08 (0.07)
	Far 2^nd^	5.80 (2.13)	0.85 (0.11)	0.06 (0.07)	0.10 (0.09)
Experiment 3	Near 1^st^	5.02 (1.40)	0.77 (0.13)	0.11 (0.08)	0.12 (0.11)
	Far 1^st^	5.28 (1.47)	0.80 (0.14)	0.09 (0.08)	0.11 (0.11)
	Near 2^nd^	6.91 (3.15)	0.84 (0.09)	0.05 (0.05)	0.11 (0.09)
	Far 2^nd^	5.95 (1.96)	0.84 (0.08)	0.04 (0.03)	0.11 (0.08)
Experiment 4	Near 1^st^	5.03 (1.67)	0.85 (0.10)	0.06 (0.05)	0.09 (0.07)
	Far 1^st^	5.18 (1.14)	0.88 (0.10)	0.05 (0.06)	0.07 (0.06)
	Near 2^nd^	5.33 (1.82)	0.88 (0.06)	0.07 (0.04)	0.05 (0.05)
	Far 2^nd^	5.79 (2.29)	0.86 (0.08)	0.05 (0.04)	0.08 (0.08)
Experiment 5	Near 1^st^	4.89 (1.59)	0.76 (0.13)	0.13 (0.10)	0.12 (0.11)
	Far 1^st^	5.69 (2.16)	0.78 (0.11)	0.09 (0.07)	0.13 (0.10)
	Near 2^nd^	6.10 (2.54)	0.81 (0.12)	0.09 (0.06)	0.11 (0.11)
	Far 2^nd^	5.80 (2.06)	0.81 (0.14)	0.08 (0.06)	0.11 (0.12)

The probability of correctly reporting the target (*P*_T_) did not differ between conditions (*t*(16) = −0.07, *p* = 0.944, Cohen’s d: −0.018). The probability of incorrectly reporting the non-target (*P*_NT_) was significantly greater for the Near condition (*t*(16) = 4.20, *p* = 0.001, Cohen’s d: 1.259). The probability of guessing (*P*_U_) was greater in the Far condition, (*t*(16) = −2.28, *p* = 0.037, Cohen’s d: −0.560). This is discussed further in the General Discussion.

### Model comparison

For model 1 we summed the log likelihood (LL), collapsed across participants, which resulted in an LL of −11946.4. Next, we used the same fitting procedure, but allowed the *P*_NT_ parameter to vary between the Near and Far conditions (Model 2). This first alternative hypothesis, allowed for the possibility that the only difference between the proximity conditions was the proportion of non-target responses. The resulting summed log likelihood was −11923.3. Lastly, in Model 3, both the *P*_NT_ and precision parameter values were allowed to vary. The resulting summed log likelihood was −11914.4.

These log likelihood values indicate that Model 2 provided a better model fit than Model 1, reflecting more non-target responses (i.e. higher *P*_NT_) for the Near than the Far condition (Table 2, which also shows parameter estimates for *P*_T_, *P*_NT_ and *P*_U_). Furthermore, Model 3 provided a better model fit than Model 2, reflecting the lower precision for the Near condition than the Far condition (Fig. 3). To determine whether these differences in log likelihood were statistically significant, a permutation test was conducted on the magnitude of the differences in summed LL between the three models. First, the data were permuted between the Near and Far conditions, collapsed across participants. These permuted data sets were submitted to the same model fitting procedure described above for all three mixture models in the hierarchy. Over repeated permutations, we produced a null distribution of the expected difference between the LL values for Model 1 and Model 2. The same procedure was then used to obtain a null distribution of expected LL differences between Models 2 and 3. We compared the empirically observed differences in LL to these null distributions. In 1000 permutations, no permutation produced a greater difference than the observed difference for either of these model comparisons (i.e. Model 2 versus Model 1 and Model 3 versus Model 2), which can conservatively be stated as p < 0.001. Thus, the model comparison revealed that there were significantly more non-target responses for the Near than the Far condition. Furthermore, from the significant difference between Model 3 and Model 2 we can conclude that, even when allowing the proportion of non-target responses to vary between conditions, WM precision was significantly lower in the Near than the Far condition.

**Table 2:** Estimates from the mixture models for Experiments 1 calculated using a model in which neither K nor P_NT_ could differ between Near and Far conditions (Model 1), a model in which only P_NT_ could differ (Model 2) and a model in which both K and P_NT_ could differ (Model 3). The 95% confidence interval is shown in parentheses, which was found by bootstrapping the data 100 times. LL denotes the log likelihood.

	K	*P*_T_	*P*_NT_	*P*_U_	LL
Model 1	4.85 (0.023)	0.83 (0.001)	0.05 (0.001)	0.12 (0.002)	−11944.40 (22.2)
Model 2	4.86 (0.022)	0.83 (0.001)	Near: 0.08 (0.001) Far: 0.03 (0.001)	0.12 (0.002)	−11921.32 (21.9)
Model 3	Near: 4.52 (0.025) Far: 5.28 (0.035)	0.83 (0.001)	Near: 0.07 (0.001) Far: 0.03 (0.001)	0.12 (0.001)	−11912.14 (22.0)

## Discussion

The greater number of non-target responses in the Near condition is consistent with a previous claim that competition causes errors in binding^21, 23^. Importantly, however, our findings indicate that the influence of competition extends to the measure of WM precision. We show, for the first time, that items presented within close spatial proximity are reported with lower WM precision, presumably due to greater competition within the visual cortex.

## Experiment 2

The results of Experiment 1 indicates that competition affects the precision of an item, and increases the probability of mis-binding. It is not clear whether competition is a purely perceptual phenomenon, occurring only when both items are presented together, or whether items that are held in WM can compete. While higher-order neural areas, including the prefrontal, temporal, and parietal cortices, are involved in WM maintenance^28–34^, according to the sensory recruitment hypothesis of WM see ref. 35, early visual areas involved in perception, including area V1, also support the active maintenance of visual information in WM. Imaging studies e.g. refs 10 and 11 have provided support for this hypothesis. We reasoned that if neurons within the same RF are recruited for the active maintenance of multiple items, as well as the encoding of items that are being perceived, then the effects of competition may still be seen even when the competing items are not presented simultaneously. This would challenge the assumption that items which are presented sequentially are free from the effects of competition e.g. refs 4, 5, 8 and 9.

In Experiment 2, we manipulated the spatial proximity between two items that were presented sequentially. We also examined whether the order of presentation influenced the extent to which an item was subject to competitive effects.

## Method

In each trial, participants were asked to retain two sequentially presented items in WM (Stimulus 1 was followed by Stimulus 2). In the Near condition, stimuli were positioned so that Stimulus 2 appeared 0.25° from the location of Stimulus 1 (centre to centre). In the Far condition, Stimulus 2 appeared 6.70° from the location of Stimulus 1 (centre to centre).

### Participants

Twenty-seven students from the University of Birmingham gave informed consent to participate in the experiment. Participants were compensated for their time, had normal or corrected-to-normal vision, and passed the Farnsworth Munsell Dichotomous D15 Colour Vision Test. One participant withdrew during the testing procedure. We applied the same outlier criteria as in Experiment 1. This left 22 participants (16 females, 6 males; mean age = 20.5 years, range = 18–30 years).

### Stimuli and Design

Stimuli were presented using the same equipment as in Experiment 1. On each trial, participants were asked to encode two squares (0.21° × 0.21°) appearing sequentially for 250 ms each, on an invisible circle with a radius of 5.50°. Both squares always appeared in the same quadrant of the screen, and the second square appeared at the instant the first square disappeared. The two squares were either 0.25° apart (centre-to-centre; contained within 0.60°; Near condition), or 6.70° apart (centre-to-centre; contained within 7.00°; Far condition). The quadrant in which the squares appeared was varied pseudo-randomly, and counterbalanced across conditions.

**Figure 4:**
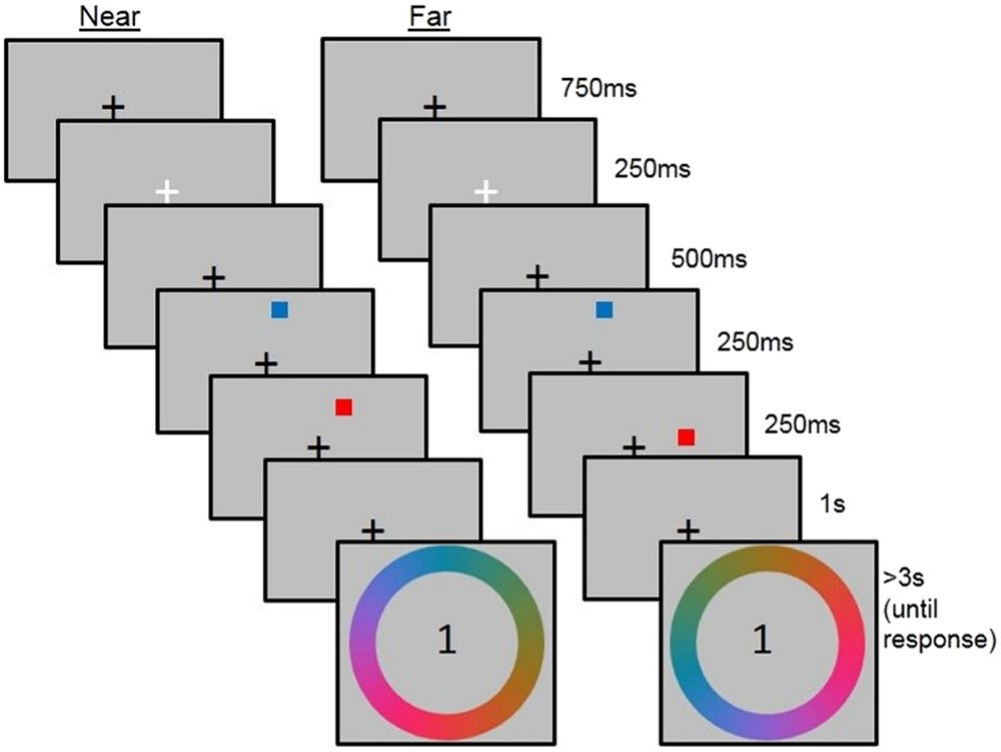
The trial sequence for the Near and Far conditions of Experiment 2.

**Figure 5:**
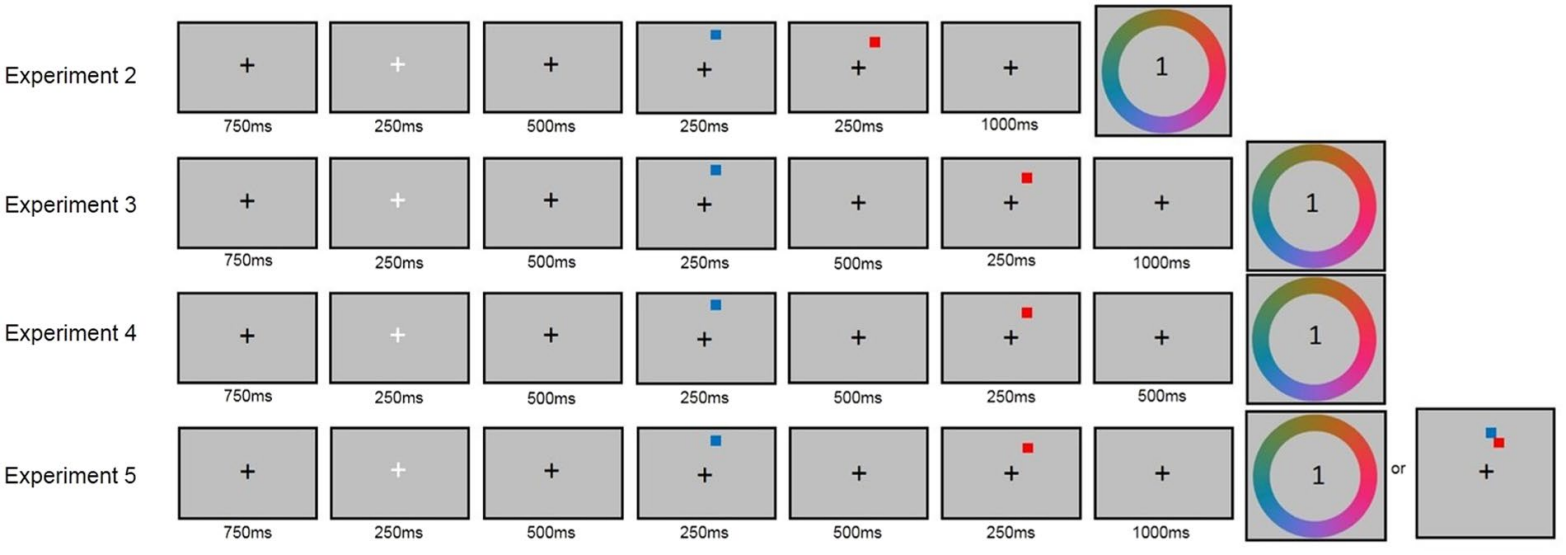
The trial sequence for the Near conditions of Experiments 2–5.

Participants were asked to indicate the colour of one of the squares (the target) by clicking on the colour wheel. Either the first or second item was cued by the presentation of the number one or two in the centre of the colour wheel. As with Experiment 1, colour allocation was pseudo-random, such that for each pair of squares, the colours differed by 60 colour units (120° on the colour wheel) and the colour distance between items was the same across all trials. The same colour pairs were used for stimuli in the Near and Far conditions, to avoid any confound associated with the choice of colours. Following 30 practice trials, participants completed 192 experimental trials (half were Near trials and half were Far trials, presented in a randomised and unpredictable order). The experiment was divided into four equal length experimental blocks, separated by self-paced breaks. The trial sequence for Near and Far conditions is shown in Fig. 4 (and also Fig. 5, for comparison with the subsequent experiments).

### Procedure

Participants were required to fixate on the fixation cross until they were asked to make a response. Each trial began with a black fixation cross (presented for 750 ms), which turned white for 250 ms to prepare participants for the onset of the first stimulus to be encoded. The first stimulus was presented for 250 ms and was immediately followed by the onset of the second stimulus, which was also presented for 250 ms. This was followed by a 1000 ms delay period, during which time a black fixation cross was displayed. Participants then saw a colour wheel and were cued to report the colour of the first or second stimulus by the number one or two, which was presented inside the colour wheel. All other procedures were maintained from Experiment 1.

## Results

**Figure 6:**
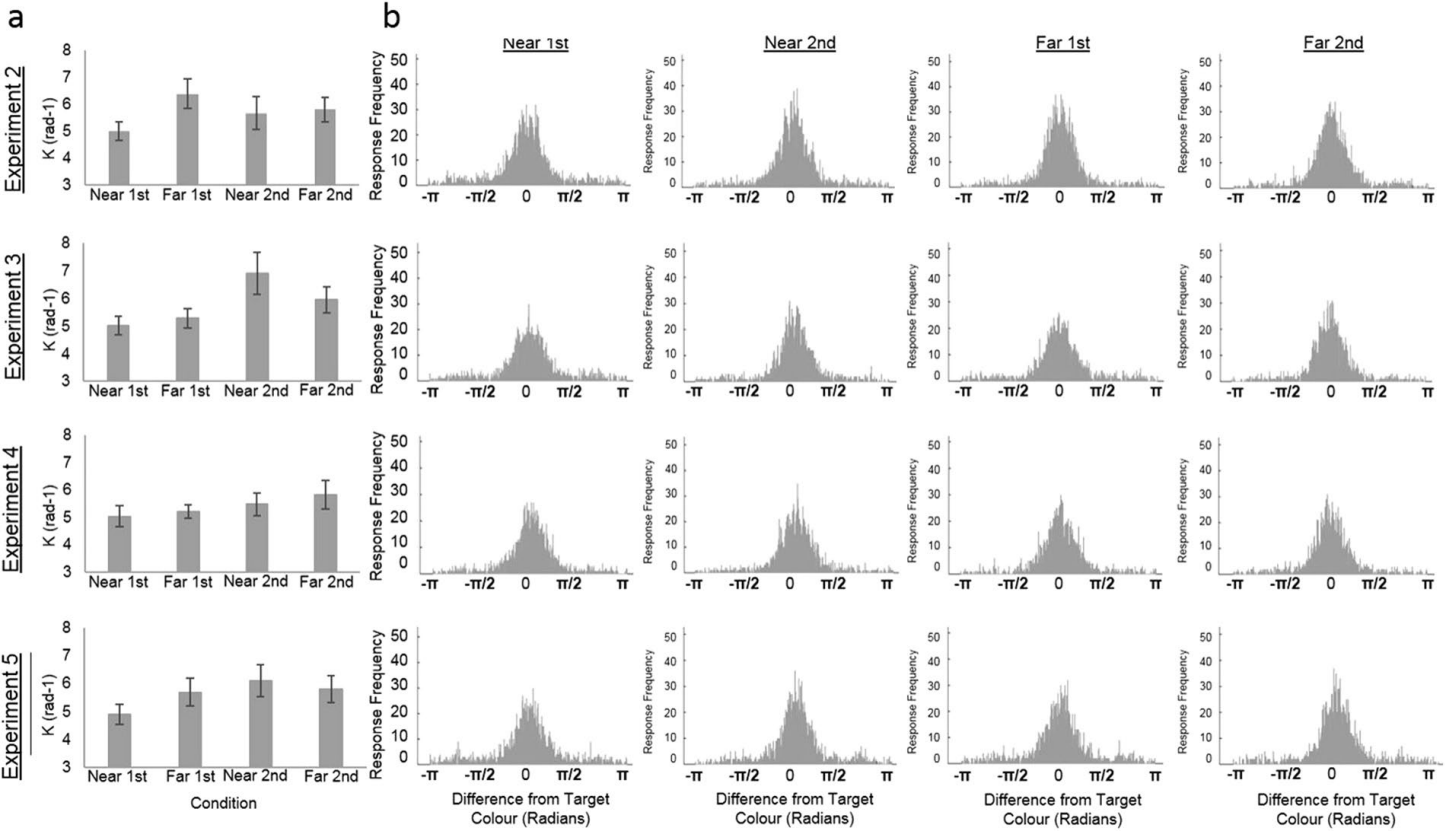
(**a**) The mean precision estimates (K) for each condition of Experiments 2–5, calculated using the mixture model^15^. Error bars represent the standard error. *Indicates p < 0.05 for paired t-tests between Near and Far conditions. (**b**) Distribution of responses relative to the target colour for the Near-First stimulus, Far- First stimulus, Near-Second stimulus and Far-Second stimulus conditions for Experiments 2–5.

Figure 6 shows the mean precision estimates (Fig. 6a) and the distribution of responses relative to the target colour (Fig. 6b) for each condition of Experiments 2–5.

### Precision

The ANOVA revealed a significant main effect of proximity (*F*(1, 21) = 6.65, *p* = 0.018, ηp^2^ = 0.241), with lower precision for Near trials. There was no main effect of order (*F*(1, 21) = 0.01, *p* = 0.918, ηp^2^ = 0.001), and no significant interaction between order and proximity, (*F*(1, 21) = 1.84, *p* = 0.190, ηp^2^ = 0.080).

### *P*_T_, *P*_NT_ and *P*_U_

Mean values for the probability parameters, *P*_T,_ *P*_NT,_ and *P*_U_ are shown in Table 1.

For the probability of correctly reporting the target, *P*_T_, there was a significant main effect of proximity (*F*(1, 21) = 10.77 *p* = 0.004, ηp^2^ = 0.339), with lower *P*_T_ for Near than Far trials. There was no significant main effect of order (*F*(1, 21) = 1.40, *p* = 0.25, ηp^2^ = 0.063). The interaction between proximity and order just failed to reach significance (*F*(1, 21) = 4.08, *p* = 0.056, ηp^2^ = 0.163. We further investigated this near-significant interaction. There was a significant difference in *P*_T_ between Near and Far conditions for the first item (paired t-test: *t*(21) = −4.49, *p* < 0.001) but not the second item, (paired t-test: *t*(21) = −0.33, *p* = 0.748).

For the probability of incorrectly reporting the non-target, *P*_NT_, there was a significant main effect of proximity (*F*(1, 20) = 17.66, *p* < 0.001, ηp^2^ = 0.457), with greater *P*_NT_ for Near compared to Far trials. There was no significant main effect of order (*F*(1, 21) = 1.94, *p* = 0.178, ηp^2^ = 0.084), but there was a significant interaction between proximity and order (*F*(1, 21) = 5.01, *p* = 0.036, ηp^2^ = 0.193). There was a greater difference between the Near and Far conditions for the first item (paired t-test: *t*(21) = 4.06, *p* = 0.001), compared to the second item (paired t-test: *t*(21) = 2.02, *p* = 0.056). This indicates that mis- binding was greater for the Near condition, particularly when the item was first in the trial.

For the probability of guessing, *P*_U_, there was no significant main effect of proximity (*F*(1, 21) = 0.78, *p* = 0.389, ηp^2^ = 0.036), no significant main effect of order (*F*(1, 21) = 0.003, *p* = 0.955, ηp^2^ < 0.01) and no significant interaction between proximity and order, (*F*(1, 21) = 0.08, *p* = 0.780, ηp^2^ = 0.004). Unlike Experiment 1, from which we observed greater values of *P*_U_ for the Far condition, Experiment 2 found no difference between Near and Far conditions. This supports the idea that the difference in *P*_U_ observed for the Far condition of Experiment 1 may have been due to participants splitting their attention between the two far-apart regions of space.

### Model comparison

To confirm the above results, model comparison was used again. The model comparison was run separately for items presented first in the trial and items presented second in the trial, to determine the effects of proximity. The parameters and fits are displayed in Table 3. For the first item, Model 2 gave a significantly better fit than Model 1 (p < 0.001) and Model 3 gave a significantly better fit compared to Model 2 (p < 0.001). However, for the second item, while Model 2 gave a significantly better fit than Model 1 (p < 0.05), there was no significant difference between Model 3 and Model 2. Thus, letting the precision and *P*_NT_ values differ between Near and Far conditions gave the best fit to the data, but only for the first item, which replicates results of our ANOVA approach.

**Table 3:** Estimates from the mixture models for Experiment 2, calculated using a model in which neither K nor P_NT_ could differ between Near and Far conditions (Model 1), a model in which only P_NT_ could differ (Model 2) and a model in which both K and P_NT_ could differ (Model 3). The 95% confidence interval is shown in parentheses, which was found by bootstrapping the data 100 times. LL denotes the log likelihood.

		K	*P*_T_	*P*_NT_	*P*_U_	LL
Second item	Model 1	5.2 (0.04)	0.84 (0.002)	0.06 (0.001)	0.10 (0.002)	−4552.8 (13.6)
	Model 2	5.2 (0.03)	0.84 (0.002)	Near: 0.07 (0.001) Far: 0.05 (0.001)	0.10 (0.002)	−4550.1 (13.8)
	Model 3	Near: 5.2 (0.05) Far: 5.3 (0.05)	0.84 (0.001)	Near: 0.07 (0.002) Far: 0.05 (0.001)	0.10 (0.002)	−4549.6 (13.8)
First item	Model 1	5.17 (0.03)	0.81 (0.002)	0.09 (0.001)	0.10 (0.002)	−4791.9 (12.4)
	Model 2	5.16 (0.03)	0.81 (0.002)	Near: 0.13 (0.002) Far: 0.06 (0.001)	0.10 (0.002)	−4770.6 (12.2)
	Model 3	Near: 4.77 (0.04) Far: 5.62 (0.05)	0.81 (0.002)	Near: 0.13 (0.002) Far: 0.06 (0.001)	0.10 (0.002)	−4765.9 (12.3)

## Discussion

The greater number of non-target responses and reduced precision in the Near condition, now observed with sequential stimulus presentation, is consistent with the findings of Experiment 1, which used simultaneous presentation. Importantly, the findings of Experiment 2 indicate that simultaneous presentation is not a requirement for competition. This extends previous work which shows greater competition for simultaneous compared to sequential presentation, but has not examined whether competition also occurs within this sequential “control” condition^4, 5, 8, 9^.

While there was a main effect of proximity upon *P*_T_, there was also a significant interaction between proximity and the order in which the items were presented. Participants were most likely to mistakenly report the non-target when the first item was probed in the Near condition. Since the second stimulus was presented immediately after the offset of the first stimulus, perceptual processing of the first stimulus may still have been ongoing when the second stimulus was presented. Our findings indicate that the representation of the first item is more likely to be disrupted when the second item is presented in close proximity. Interestingly, the difference between Near and Far conditions just failed to reach statistical significance for the second stimulus.

The competitive effects reported here may arise in iconic memory, with the perception of a second item distorting the processing or consolidation of the first item. To determine whether items held within WM (and no longer in iconic memory) can contribute to, or be susceptible to competition, we added a 500 ms inter-stimulus interval (ISI) between the presentation of the two stimuli.

## Experiment 3

In Experiment 3 we introduced a 500 ms ISI between Stimulus 1 and Stimulus 2. If the competition we observed in Experiments 1 and 2 requires both items to be perceived together, or for at least one of the items to be held in iconic memory, then we should see equivalent WM performance for the Near and Far conditions in Experiment 3. If items held in WM can also compete, then we would expect to replicate the results of Experiments 1 and 2.

## Method

### Participants

Twenty-one students from the University of Birmingham (none of whom had participated in Experiments 1 and 2) gave informed consent to participate in Experiment 3. One participant withdrew after the practice block. Data from three participants were removed due to values for at least one of the model components in any of the four conditions being greater than 3 standard deviations from the group mean. This left 17 participants (11 females, 6 males, mean age = 23.4 years; range = 19–34 years).

### Stimuli and Design

The same stimuli were used as in Experiment 2. Following 30 practice trials, participants completed 192 experimental trials.

### Procedure and Data analysis

The experimental procedure and data analysis were the same as for Experiment 2, with the addition of a 500 ms delay period between the offset of Stimulus 1 and the onset of Stimulus 2 (see Fig. 5). The distributions of responses were analysed in the same way as in Experiments 1–3.

## Results

Figure 6 shows the mean precision estimates (Fig. 6a) and the distribution of responses relative to the target colour (Fig. 6b) for each condition of Experiments 2–5.

### Precision

The results of the ANOVA indicate that there was no significant main effect of proximity (*F*(1, 16) = 0.74, *p* = 0.404, ηp^2^ = 0.044) upon WM precision. There was a significant main effect of order (*F*(1, 16) = 6.23, *p* = 0.024, ηp^2^ = 0.280), with greater precision for Stimulus 2, but no interaction between order and proximity, (*F*(1, 16) = 2.51, *p* = 0.133, ηp^2^ = 0.136).

We had observed a significant main effect of proximity for Experiment 2. An ANOVA with factors Experiment (Experiment 2 or Experiment 3), order and proximity revealed a significant interaction between proximity and Experiment (*F*(1, 37) = 5.15, *p* = 0.029, ηp^2^ = 0.122). This demonstrates that proximity affected precision when Stimulus 2 immediately followed Stimulus 1 (Experiment 2), but not when there was a 500 ms gap between the two stimuli (Experiment 3).

### *P*_T_, *P*_NT_ and *P*_U_

Mean values for the probability parameters, *P*_T,_ *P*_NT,_ and *P*_U_ are shown in Table 1.

For the probability of correctly reporting the target, *P*_T_, the main effect of proximity (*F*(1, 16) = 1.18 *p* = 0.293, ηp^2^ = 0.069) was not significant. The main effect of order was significant (*F*(1, 16) = 10.659, *p* = 0.005, ηp^2^ = 0.400). In contrast to Experiment 2, the interaction between order and proximity was not significant (*F*(1, 16) = 1.14, *p* = 0.302, ηp^2^ = 0.066). The difference in the effect of proximity between Experiments 2 and 3 did not reach significance (the experiment by proximity interaction was not significant; *F*(1, 37) = 0.457, *p* = 0.503, ηp^2^ = 0.012.

For the probability of incorrectly reporting the non-target, *P*_NT_, there was no significant main effect of proximity (*F*(1, 16) = 2.397, *p* = 0.141, ηp^2^ = 0.130). There was a main effect of order (*F*(1, 16) = 15.24, *p* = 0.001, ηp^2^ = 0.488, and there was no significant interaction between proximity and order (*F*(1, 16 = 0.498, *p* = 0.490, ηp^2^ = 0.030). This significantly differed from Experiment 2, whereby a mixed ANOVA with experiment as a between group factor revealed a significant interaction between proximity and experiment upon *P*_NT_, (*F*(1, 37) = 5.21, *p* = 0.028, ηp^2^ = 0.123), demonstrating that proximity affects the probability of incorrectly reporting the non- target when Stimulus 2 immediately followed Stimulus 1, but not in Experiment 3.

For the probability of guessing, *P*_U_ there was no significant main effect of proximity (*F*(1, 16) = 0.109, *p* = 0.745, ηp^2^ = 0.007) and no significant main effect of order (*F*(1, 16) = 0.079, *p* = 0.782, ηp^2^ = 0.005). There was no significant interaction between order and proximity (*F*(1, 16) = 0.124, *p* = 0.729, ηp^2^ = 0.008).

### Model comparison

The same model comparison process used in Experiment 2 was used for Experiment 3. The parameters and fits are displayed in Table 4. For the first item, Model 2 gave a significantly better fit than Model 1 (p < 0.05), but there was no significant difference between Model 3 and Model 2 (p = 0.17). For the second item, Model 2 was not significantly better than Model 1 (p = 0.59) and Model 3 was not significantly better than Model 1 (p = 0.496). Therefore, proximity had minimal effects on precision and *P*_NT_.

**Table 4:** Estimates from the mixture models for Experiment 3, calculated using a model in which neither K nor P_NT_ could differ between Near and Far conditions (Model 1), a model in which only P_NT_ could differ (Model 2) and a model in which both K and P_NT_ could differ (Model 3). The 95% confidence interval is shown in parentheses, which was found by bootstrapping the data 100 times. LL denotes the log likelihood.

		K	*P*_T_	*P*_NT_	*P*_U_	LL
Second item	Model 1	5.0 (0.04)	0.87 (0.001)	0.06 (0.001)	0.07 (0.002)	−3761.6 (12.7)
	Model 2	5.0 (0.04)	0.87 (0.001)	Near: 0.07 (0.002) Far: 0.06 (0.001)	0.07 (0.002)	−3760.8 (12.7)
	Model 3	Near: 5.0 (0.04) Far: 5.0 (0.05)	0.87 (0.001)	Near: 0.07 (0.002) Far: 0.06 (0.001)	0.07 (0.002)	−3760.3 (12.7)
First item	Model 1	4.9 (0.03)	0.86 (0.002)	0.05 (0.001)	0.09 (0.002)	−3881.5 (12.1)
	Model 2	4.9 (0.03)	0.86 (0.002)	Near: 0.06 (0.002) Far: 0.04 (0.001)	0.09 (0.002)	−3880.1 (12.1)
	Model 3	Near: 4.67 (0.05) Far: 5.1 (0.05)	0.86 (0.002)	Near: 0.06 (0.002) Far: 0.04 (0.001)	0.09 (0.002)	−3878.5 (12.1)

## Discussion

In Experiment 2, we demonstrated reduced precision and *P*_T_ and increased *P*_NT_ in the Near condition, particularly for the first item in the sequence. In Experiment 3, with a 500 ms gap between Stimulus 1 and Stimulus 2, the effect of competition was eliminated. This might indicate that items were competing within perception in Experiment 2, with the first stimulus still in iconic memory. It might also suggest that items held within WM are better protected from competition with incoming information than items held within iconic memory. However, as we go on to explain, the results of Experiment 5 indicate that the effects of competition on items held in WM are task-dependent, and that with certain task conditions, representations held within WM may also be affected by competition.

## Experiment 4

Experiment 1 revealed that competition affects the number of mis-binding errors and the precision of an item within WM. In Experiment 2 we observed this effect when the stimuli are presented sequentially. The results of Experiment 3 suggest that this might arise only during perception, when the first item is still in iconic memory. In Experiment 4 we reduced the period between the offset of Stimulus 2 and the colour wheel to 500 ms. One possibility is that with the addition of a 500 ms delay period in Experiment 3, the first item had been in WM for too long to see an effect of competition on precision. Inserting a 500 ms interval from the offset of Stimulus 2 to the response period allowed us to equate the overall time between the onset of Stimulus 1 and the response period with Experiment 2.

## Method

### Participants

Twenty students from the University of Birmingham (none of whom had participated in Experiments 1–3) gave informed consent to participate in Experiment 4. Data from 1 participant were removed due to values for at least one of the model components in any of the four conditions being greater than 3 standard deviations from the group mean. This left 19 participants (mean age = 25 years; range = 20–32 years).

### Stimuli, Design, Procedure and Data Analysis

The stimuli and procedure replicated those of Experiments 3, but there was 500 ms instead of 1000 ms between the offset of Stimulus 2 and the probe, during which time a fixation cross was shown (see Fig. 5). Following 30 practice trials, participants completed 192 experimental trials. The distribution of responses was analysed in the same way as in Experiments 1–3.

## Results

Figure 6 shows the mean precision estimates (Fig. 6a) and the distribution of responses relative to the target colour (Fig. 6b) for each condition of Experiments 2–5.

### Precision

Figure 6a shows the mean precision estimates for each condition. There was no significant main effect of proximity (*F*(1, 18) = 0.84, *p* = 0.37, ηp^2^ = 0.045) upon WM precision, and no significant main effect of order (*F*(1, 18) = 1.62, *p* = 0.219, ηp^2^ = 0.083). There was no significant interaction between order and proximity (*F*(1, 18) = 0.31, *p* = 0.584, ηp^2^ = 0.017).

### *P*_T_, *P*_NT_ and *P*_U_

Mean values for the probability parameters, *P*_T,_ *P*_NT,_ and *P*_U_ are shown in Table 1. For the probability of correctly reporting the target, *P*_T_, there was no significant main effect of proximity (*F*(1, 18) = 0.10 *p* = 0.758, ηp^2^ = 0.005) and no significant main effect of order (*F*(1, 18) = 0.14, *p* = 0.717, ηp^2^ = 0.007). There was an almost significant interaction between proximity and order (*F*(1, 18) = 4.38, *p* = 0.051, ηp^2^ = 0.196). Although not significant, *P*_T_ was greater for the Far compared to the Near condition when Stimulus 1 was probed (paired t-test: *t*(18) = −1.93, *p* = 0.069), but greater for the Near condition when Stimulus 2 was probed (although this difference was far from reaching significance, paired t-test: *t*(18) = 0.99, *p* = 0.337). This pattern of results replicates that of Experiment 2, where *P*_T_ was also reduced for the first item in the Near condition.

For the probability of incorrectly reporting the non-target, *P*_NT_, in contrast to Experiments 1–2, there was no significant main effect of proximity (*F*(1, 18) = 1.57, *p* = 0.226, ηp^2^ = 0.08), and no significant main effect of order (*F*(1, 18) = 0.48, *p* = 0.497, ηp^2^ = 0.026). There was no significant interaction between proximity and order (*F*(1, 18) = 0.93, *p* = 0.347, ηp^2^ = 0.049).

For the probability of guessing, *P*_U_ there was no significant main effect of proximity (*F*(1, 18) = 0.14, *p* = 0.711, ηp^2^ = 0.008), and no significant main effect of order (*F*(1, 18) = 0.884, *p* = 0.360, ηp^2^ = 0.047). The interaction between proximity and order was close to reaching significance (*F*(1, 18) = 4.27, *p* = 0.054, ηp^2^ = 0.192). Although not significantly different, *P*_U_ was greater for the Near condition compared to the Far condition when Stimulus 1 was probed (paired t-test: *t*(18) = 1.42, *p* = 0.17), but *P*_U_ was greater in the Far condition compared to the Near condition when Stimulus 2 was probed (paired t-test: *t*(18) = −1.43, *p* = 0.17).

### Model comparison

The same model comparison process used in Experiments 2 and 3 was used for Experiment 4. The parameters and fits are displayed in Table 5. For the first item, Model 2 did not give a significantly better fit than Model 1 (p = 0.1) and Model 3 did not give a significantly better fit than Model 2 (p = 0.19). For the second item, Model 2 did not give a significantly better fit than Model 1 (p = 0.84) and Model 3 did not give a significantly better fit than Model 1 (p = 0.36). Therefore, there was no effect of proximity on precision and *P*_NT_ for Experiment 4.

**Table 5:** Estimates from the mixture models for Experiment 4, calculated using a model in which neither K nor P_NT_ could differ between Near and Far conditions (Model 1), a model in which only P_NT_ could differ (Model 2) and a model in which both K and P_NT_ could differ (Model 3). The 95% confidence interval is shown in parentheses, which was found by bootstrapping the data 100 times. LL denotes the log likelihood.

		K	*P*_T_	*P*_NT_	*P*_U_	LL
Second item	Model 1	5.8 (0.05)	0.83 (0.001)	0.05 (0.001)	0.12 (0.002)	−3410.3 (12.5)
	Model 2	5.8 (0.05)	0.83 (0.002)	Near: 0.05 (0.001) Far: 0.04 (0.001)	0.12 (0.002)	−3409.6 (12.5)
	Model 3	Near: 6.0 (0.06) Far: 5.7 (0.06)	0.83 (0.002)	Near: 0.05 (0.001) Far: 0.04 (0.001)	0.12 (0.002)	−3408.6 (12.6)
First item	Model 1	4.9 (0.04)	0.78 (0.002)	0.10 (0.001)	0.12 (0.003)	−3954.5 (11.7)
	Model 2	4.9 (0.04)	0.78 (0.002)	Near: 0.11 (0.002) Far: 0.08 (0.001)	0.12 (0.003)	−3952.3 (11.7)
	Model 3	Near: 4.7 (0.05) Far: 5.1 (0.05)	0.78 (0.002)	Near: 0.11 (0.002) Far: 0.08 (0.001)	0.12 (0.003)	−3950.9 (11.6)

## Discussion

As with Experiment 3, we failed to find a difference between Near and Far conditions in Experiment 4. Our findings indicate that the precision and mis-binding effects we observed in Experiments 1–2 may be due to perceptual competition. This includes competition between simultaneously presented items (Experiment 1), and sequential presentation with Stimulus 1 still remaining in iconic memory when Stimulus 2 is shown (Experiment 2). Alternatively, as information about the location of the stimuli was not relevant to the task, participants may have failed to maintain this information in WM, reducing or removing the effects of competition.

## Experiment 5

In Experiment 5, we encouraged participants to maintain information about the location of the stimuli. Participants were asked to make a location-based judgment on 25% of the trials, instead of performing the colour wheel task. To perform well on the location-based task, participants must maintain the precise location of the stimuli in WM. This was done to address the possibility that items held in WM can be affected by competing proximal stimuli if their spatial position is maintained in WM, and that we had failed to see the effects of competition in Experiments 3 and 4 because their spatial position had not been maintained.

## Method

### Participants

Twenty seven students from the University of Birmingham (none of whom had participated in Experiments 1–4) gave informed consent to participate in Experiment 5. Two participants withdrew during the experimental procedure. Data from 6 participants were removed due to values for at least one of the model components in any of the four conditions being greater than 3 standard deviations from the group mean, or due to the participant responding to the target on less than 50% of the trials. This left 19 participants (mean age = 19 years; range = 18–23 years).

### Stimuli and Design

The stimuli and presentation times replicated those of Experiment 3. There was a 1000 ms delay period between the offset of Stimulus 2 and the response phase. There were two tasks; 1) the colour report task in which participants reported the colour of one of the two stimuli on a colour wheel, as used in Experiments 2–4, and 2) a location judgment task. For the colour report task, there were 384 trials; 192 were Near condition trials, 192 were Far condition trials. For the location judgment trials there were 128 trials. At the response phase, participants were presented with the two squares simultaneously. The colours of the two squares matched the colours used at presentation. Participants were asked to report whether the location of either of the squares had changed. For 32 trials, neither of the squares changed location, for 32 trials both of the squares changed location and for 64 trials just one of the squares changed location. Participants indicated “change” or “no change” by pressing buttons 1 or 2 on the keyboard. There were 30 practice trials, composed of both colour report and location judgement trials.

### Procedure

The experimental procedure for Experiment 3 was maintained, except that participants in Experiment 5 were asked to perform the colour report task when they saw the colour wheel during the response phase and the location judgement (change detection) task when they saw the two squares presented during the response phase. Participants did not know which task they would be asked to perform until the response phase. Figure 6 shows the trial sequence for the Near condition.

### Data Analysis

The distribution of responses was analysed in the same way as in Experiments 1–4.

## Results

Figure 6 shows the mean precision estimates (Fig. 6a) and the distribution of responses relative to the target colour (Fig. 6b) for each condition of Experiments 2–5.

### Precision

The results of the ANOVA indicate that there was no significant effect of proximity (*F*(1, 18) = 1.42, *p* = 0.249, ηp^2^ = 0.073) upon WM precision, and no significant effect of order (*F*(1, 18) = 2.81, *p* = 0.111, ηp^2^ = 0.135). There was no significant interaction between order and proximity (*F*(1, 18) = 1.30, *p* = 0.269, ηp^2^ = 0.067).

### *P*_T_, *P*_NT_ and *P*_U_

Mean values for the probability parameters, *P*_T,_ *P*_NT,_ and *P*_U_ are shown in Table 1. For the probability of correctly reporting the target, *P*_T_, there was no significant main effect of proximity (*F*(1, 18) = 0.43 *p* = 0.521, ηp^2^ = 0.023). There was a significant main effect of order (*F*(1, 18) = 6.47, *p* = 0.020, ηp^2^ = 0.264), with a reduced probability of reporting the target for Stimulus 1. There was no significant interaction between order and proximity (*F*(1, 18) = 0.19, *p* = 0.670, ηp^2^ = 0.010).

For the probability of incorrectly reporting the non-target, *P*_NT_, there was a significant main effect of proximity (*F*(1, 18) = 4.70, *p* = 0.044, ηp^2^ = 0.207), with greater mis-binding in the Near condition. The main effect of order was close to reaching significance (*F*(1, 18) = 4.40, *p* = 0.051, ηp^2^ = 0.196), with more mis-binding of Stimulus 1 than Stimulus 2. There was no significant interaction between proximity and order *F*(1, 18) = 1.44, *p* = 0.245, ηp^2^ = 0.074). Although there was no significant interaction between proximity and order, we were keen to ascertain whether there was a significant difference between Near and Far conditions for both the first and second stimulus in the sequence. This has important implications for our interpretation. Should only the first stimulus show a significant difference, it may be that the process of encoding the second stimulus disrupts the WM representation of the first stimulus. It would therefore mean that our interpretation should not assume that each stimulus affects the others, as is believed to be the case with simultaneous competition. For this reason, we performed separate t-tests for the first and second stimulus in the sequence, despite the interaction between proximity and order not reaching significance. For Stimulus 1 there was a significant difference between Near and Far conditions (paired t-test: *t*(18) = 2.57, *p* = 0.019), but for Stimulus 2 there was no significant difference between Near and Far conditions (paired t-test: *t*(18) = 0.48, *p* = 0.636).

For the probability of guessing, *P*_U_, there was no significant main effect of proximity (*F*(1, 18) = 0.612, *p* = 0.444, ηp^2^ = 0.033) and no significant main effect of order (*F*(1, 18) = 1.17, *p* = 0.295, ηp^2^ = 0.061). There was no significant interaction between order and proximity (*F*(1, 18) = 0.07, *p* = 0.797, ηp^2^ = 0.004).

### Model comparison

The same model comparison process used in Experiments 2, 3 and 4 was used for Experiment 5. The parameters and fits are displayed in Table 6. For the first item, Model 2 gave a significantly better fit than Model 1 (p < 0.01), but Model 3 was not significantly better than Model 2 (p = 0.212). For the second item, Model 2 was not significantly better than Model 1 (p = 0.49) and Model 3 did not give a significantly better fit than Model 1 (p = 0.79). For Experiment 5, proximity had an effect on *P*_NT_ only for the first item.

**Table 6:** Estimates from the mixture models for Experiment 5, calculated using a model in which neither K nor P_NT_ could differ between Near and Far conditions (Model 1), a model in which only P_NT_ could differ (Model 2) and a model in which both K and P_NT_ could differ (Model 3). The 95% confidence interval is shown in parentheses, which was found by bootstrapping the data 100 times. LL denotes the log likelihood.

		K	*P*_T_	*P*_NT_	*P*_U_	LL
Second item	Model 1	5.5 (0.05)	0.80 (0.002)	0.08 (0.001)	0.12 (0.002)	−4104.1 (14.1)
	Model 2	5.5 (0.05)	0.80 (0.002)	Near: 0.09 (0.002) Far: 0.08 (0.002)	0.12 (0.002)	−4103.4 (14.1)
	Model 3	Near: 5.5 (0.06) Far: 5.5 (0.06)	0.80 (0.002)	Near: 0.09 (0.002) Far: 0.08 (0.002)	0.12 (0.002)	−4102.7 (14.1)
First item	Model 1	5.0 (0.04)	0.76 (0.002)	0.10 (0.002)	0.14 (0.002)	−4535.3 (11.9)
	Model 2	5.0 (0.04)	0.76 (0.002)	Near: 0.12 (0.002) Far: 0.09 (0.002)	0.14 (0.002)	−4530.8 (11.8)
	Model 3	Near: 4.7 (0.05) Far: 5.2 (0.06)	0.76 (0.002)	Near: 0.12 (0.002) Far: 0.09 (0.002)	0.14 (0.002)	−4529.4 (11.8)

## Discussion

In Experiment 5, the introduction of the location judgement task brought back the effect of competition on *P*_NT_, although no effect of competition was observed for WM precision. An effect of competition had been seen on precision with simultaneous stimulus presentation (Experiment 1) and when Stimulus 2 immediately followed Stimulus 1 (Experiment 2). However, the difference in *P*_NT_ between Near and Far conditions indicates that an effect of competition can be seen with sequential stimulus presentation, and with a delay of 500 ms between the stimuli, so that Stimulus 1 would not still have been in iconic memory when Stimulus 2 was presented. Again, we do not wish to imply that the competition occurs “within” working memory or iconic memory, and our results do not tell us when the competition occurs, but they do indicate that simultaneous presentation is not a prerequisite for competition to occur.

### General Discussion

Across five experiments, we examined the effects of competition on WM performance by manipulating the spatial proximity between items. Initially, we presented items simultaneously and demonstrated not only more mis-binding in the Near (high competition) condition, but also reduced WM precision in this condition (Experiment 1). We also examined whether spatial proximity also affected performance when stimuli were presented sequentially. When the second item immediately followed the first, spatial proximity again affected performance (specifically WM precision and the probability of reporting the non-target) (Experiment 2), indicating that competition is not restricted to conditions in which competing stimuli are presented simultaneously. Order effects also indicate that competition more strongly affected WM performance for the first stimulus. To address the possibility that these competition effects were dependent upon the first item being held in iconic memory, we introduced a delay between the two items. In Experiments 3–5, there was a 500 ms delay between the two stimuli. With 1000 ms between the offset of the second stimulus and the response period, we did not find any evidence of competition (Experiment 3). Similarly, in Experiment 4, reducing the delay between the offset of the second stimulus and the response period to 500 ms, we again saw no effect of proximity on WM performance. With Experiment 5, we introduced a second task in order to encourage maintenance of the specific location of the stimuli. Here, we observed an effect of proximity on the probability of reporting the non-target, as we had seen for simultaneous presentation (Experiments 1 and 2). These results indicate that competitive interactions between stimuli are not restricted to conditions in which the stimuli are presented together.

Although the effects of competition did not extend to the measure of WM precision in Experiment 5, we did observe an effect of competition on WM precision in Experiments 1 and 2. This also extends previous research into competition and sensory suppression during perception e.g. ref. 1, 4, 5, 36, 37. By comparing sequential versus simultaneous stimulus presentation, Ihssen *et al*.^9^ report that competition affects WM capacity, but they did not address whether the fidelity of a WM representation is affected. Emrich & Ferber^21^ and Tamber-Rosenau *et al*.^23^ manipulated spatial proximity, and observed a greater probability of reporting the non-target (which they interpret as mis-binding of stimulus features). Interestingly however, they did not find evidence for an effect of spatial proximity on WM precision, as we observed. We suggest that this discrepancy is due to the difference in the distance between the stimuli used in their studies and ours. The stimuli were closer together in our study. Critically, our findings show that the outcome measure of WM precision can be compromised by competition.

The present results apparently contradict those of Lin and Luck^24^ who observed enhanced WM performance (capacity) for similarly coloured items (the high competition condition) compared to items of dissimilar colours (the low competition condition). However, their participants were required to report the colour of remembered items, and colour was also the feature used to manipulate competition. In our experiment, the task demand (colour report) was orthogonal to the dimension being manipulated (spatial proximity). This difference may account for the discrepancy. Alternatively, perceptual averaging^38^ may explain the difference. In the study by Lin and Luck^24^, a greater likelihood of the changed item being far from the perceptual average in the high similarity condition may have made the change easier to identify, and account for superior performance in this condition.

Despite the enhanced precision associated with Far trials in Experiment 1, they were also associated with a greater probability of guessing. This difference in guessing may be attributed to a precision-capacity trade-off, as reported elsewheree.g. ref. 39. It is possible that, for some participants, attention was oriented to one item at the expense of the other, leading to more guessing (although no significant difference in *P*_T_ was observed). However, it should be noted that the estimates for *P*_U_ were low across all conditions (<0.2).

With Experiments 2 and 5 we tested for competitive effects between stimuli presented sequentially. Previously, sequential presentation has been viewed as a low/no competition control condition, following the assumption that sensory suppression among stimuli within RFs can only take place when they are perceived together^8, 9, 21, 40, 41^. As far as we are aware, no studies have manipulated spatial proximity between sequentially presented stimuli to address whether competition affects WM performance when the stimuli are not perceived together. This is a critical question given that the visual world is intrinsically continuous and dynamic.

In Experiment 2, we demonstrated that sequential presentation of stimuli presented close together in space, with the second stimulus immediately following the first, resulted in less precision and greater mis-binding, particularly when the first stimulus was probed. One possibility is that the first item was still in iconic memory when it’s processing and consolidation was disrupted by competition from the second item.

Although the effects of competition disappeared when we introduced a 500 ms delay between the first and second stimuli (Experiments 3 and 4), we again saw evidence of competition in Experiment 5, when an additional task was introduced to encourage participants to maintain the locations of stimuli in WM. In Experiment 5, we observed greater *P*_NT_ in the Near condition compared to the Far condition, but, unlike in Experiment 1, no difference in WM precision. One possibility is that we did not observe an effect of proximity on WM precision because, when perceptual competition occurs between one item held in working memory and one item being displayed, the effects are more subtle and only the effect of proximity on *P*_NT_ reaches significance. The fact that, with simultaneous presentation and a greater distance between stimuli, others have observed an effect of competition on *P*_NT_ but not precision^21, 23^, supports the idea that the effect of competition on precision is somehow weaker. Alternatively, it is possible that the effect of proximity on *P*_NT_ represents a different kind of competition to the effect of proximity on precision. Perhaps, with sequential presentation, the items are precisely represented during encoding, but that proximity affects their maintenance within working memory, even perhaps the binding of colours to locations. Further experiments are needed to address this. Also, for a direct comparison of competition effects with simultaneous and sequential stimulus presentation, further experiments will need to examine whether there are any differences in eye-movements between the two conditions and these may affect competition.

According to the sensory recruitment hypothesis of WM, early visual areas involved in perception are used to support the active maintenance of visual spatial information see ref. 35. If neurons within the same RF are recruited for the active maintenance of two proximal items, then we suggest that these items may still compete even if they are not presented together. Presumably, our location judgment task in Experiment 5 encouraged participants to maintain location information such that the effects of competition were brought back, or enhanced. Avoiding retinotopic specificity (which may have been the case in Experiments 3 and 4) presumably protected the items from competition.

These results also have important implications for formal models of working memory that simulate the interference between stored items. In Swan & Wyble^42^, a shared pool of neural resources binds the colours and locations of multiple objects by assigning them to distinct tokens. This model predicts an effect of spatial proximity on precision, since, in the model, items that are spatially closer share more of their neural representation and thus produce greater mutual interference. However, what the model does not predict is the importance of temporal separation during presentation, which is shown here to protect the items from increased interference caused by reduced inter-item distance. The implications for the model are that time plays a key role in increasing the distinctiveness between tokens, even at relatively long intervals. Previously, it had been assumed from work on the attentional blink that sequential items presented at SOAs longer than 200 ms elicited entirely distinct working memory representations by exceeding the sparing window^43, 44^, but in Experiment 2 we observed an effect of proximity with a 250 ms SOA, which challenges the idea that 200 ms is sufficient for there to be distinct WM representations.

In conclusion, we demonstrate that competition affects WM performance. Simultaneously presenting two items in close spatial proximity, so that they are likely to compete within early visual areas, results in decreased WM precision and increased mis-binding. We also demonstrate that sequential conditions are not free from competition. Our order effects suggest that the first stimuli encoded in WM is susceptible to the effects of competition from the second stimulus. With a short delay period between the two stimuli, the effects of competition are no longer evident. However, when participants are required to maintain the precise spatial location of the two stimuli, the effects of competition can be seen, with more mis-binding associated with high competition, but no difference in WM precision. The present report shows the effects of competition on WM performance both when items compete at perception and when one item is held in iconic memory or WM when the other is perceived, and extends our understanding of the limitations of human information processing. Evidence also indicates that the ability to ignore distraction involves the resolution of competitive interactions between stimuli^39, 40, 45, 46^, suggesting that our results may also have implications for the processing of task-irrelevant as well as task-relevant stimuli.
